# Ectopia cordis with endocardial cushion defect: Prenatal ultrasonographic diagnosis with autopsy correlation

**DOI:** 10.4103/0974-2069.74048

**Published:** 2010

**Authors:** K Balakumar, K Misha

**Affiliations:** Department of Ultrasonography, PVS Hospital, Kerala, India

**Keywords:** Atrioventricular canal defect, amniotic band disruption, ectopia cordis

## Abstract

The prenatal ultrasonographic diagnosis of ectopia cordis associated with a complex intra-cardiac defect (common atrium, common atrioventricular valve with single ventricle) is illustrated in a 32-week gestation fetus. The fetus showed associated features of amniotic band disruption sequence. The cardiac autopsy findings correlated with the antenatal diagnosis. The association of ectopia cordis with amniotic band disruption is rare and infrequently reported in literature.

## INTRODUCTION

The association of amniotic band disruption sequence with ecopia cordis is very rare. The antenatal ultrasonographic diagnosis of this condition is reported here. The complex cardiac anomalies were correlated with fetal autopsy.

## CASE REPORT

**Figure 1:**
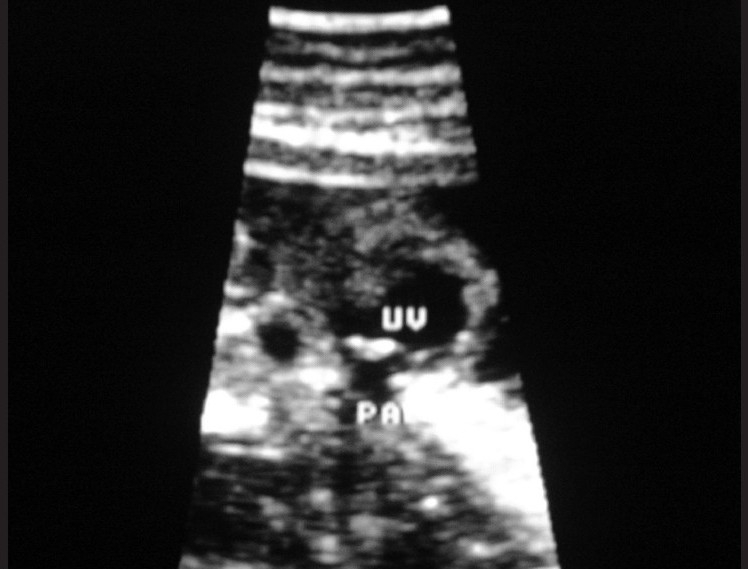
A midline sagittal section of the fetus showing the heart lying outside the thorax, with a single ventricle (UV). A segment of the pulmonary artery (PA) is visualized, characterized by a short trunk splitting into right and left branches

**Figure 2:**
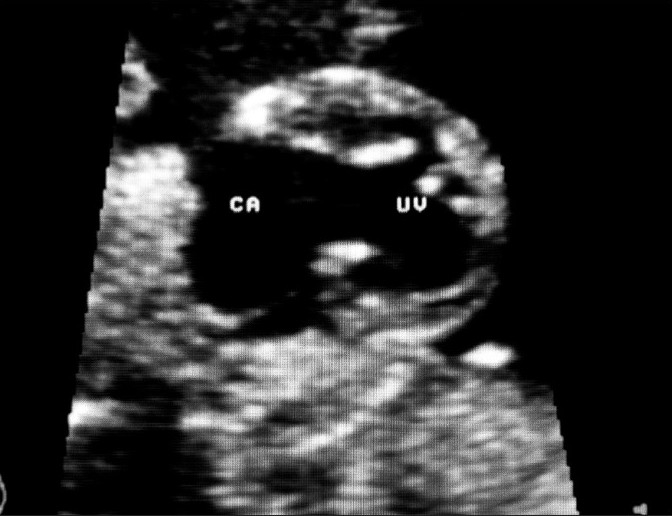
The magnified view of the fetal heart shows a common atrium (CA) and a single ventricle (UV). The dysplastic atrioventricular valves are seen in between these chambers, displaced to the sides

**Figure 3:**
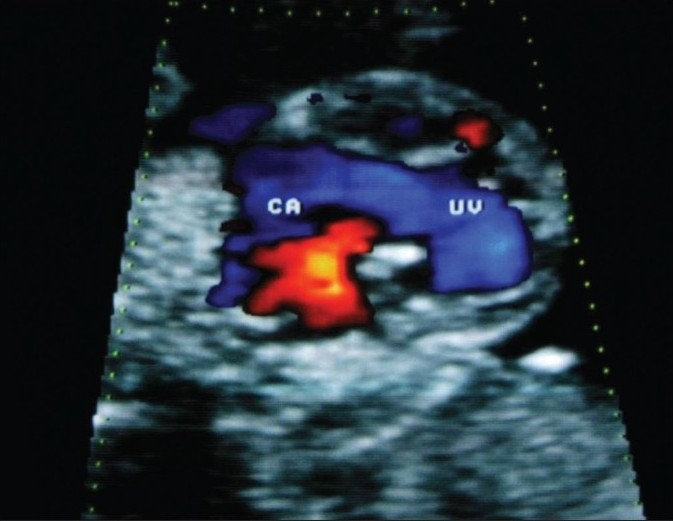
Color flow imaging shows the jet of blood passing through the widely patent atrioventricular canal connecting the common atrium (CA) and univentricle (UV)

**Figure 4:**
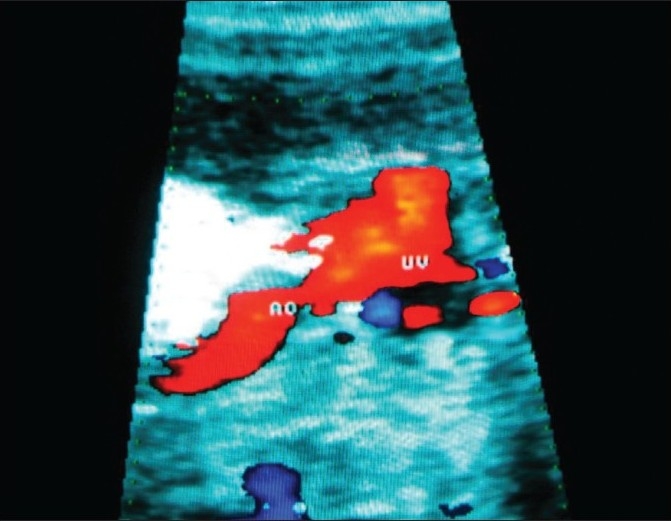
Part of aorta (AO) is traced as originating from the univentricle (UV) and continuing as the ascending aorta and as part of the arch without branching

A primigravida with 32 weeks of amenorrhea and no risk factors for congenital heart defects was referred to us for a routine cardiac screening. Fetal echocardiography performed using Aloka 5000 equipment showed features suggestive of ectopia cordis [Figure 1]. The intra-cardiac anatomy showed common atrium communicating with a single ventricle through a common atrioventricular (AV) valve [Figures 2 and 3]. Both the outflow tracts were visualized [Figures 1 and 4]. The normal orientation of the great vessels was lost. There was no evidence of hydrops fetalis. In addition, there were multiple extra-cardiac defects including skeletal and cranial abnormalities. The skeletal abnormalities included dysplastic right upper limb (all bones were shorter with satisfactory calcification) and constriction abnormalities of the digits of left upper limb. There was evidence of multiple amniotic bands attached to the abdominal viscera and brain. The brain tissue was partially herniating.

**Figure 5:**
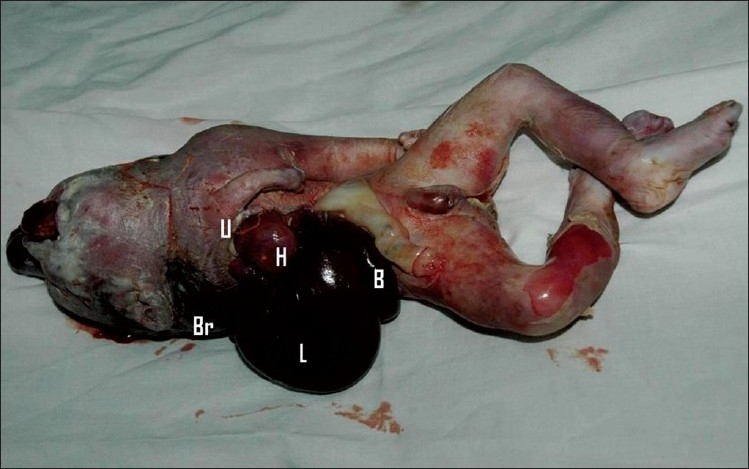
The abortus shows the heart (H) outside the chest, lying closer to the liver (L) and bowel loops (B). The deformed right upper limb (U) is seen near the heart. The brain matter (Br) is seen behind the neck

**Figure 6:**
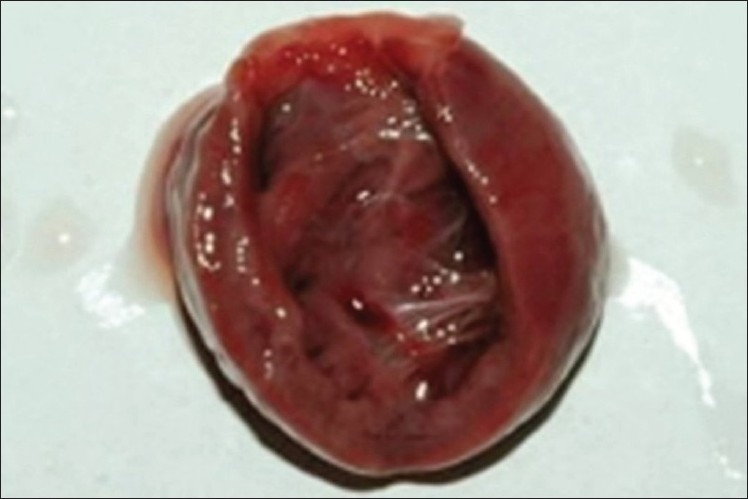
The autopsy of the fetal heart shows the continuity between the common atrial and ventricular chambers. The whole heart appears like a large chamber with trabeculae

The parents opted for therapeutic abortion. The autopsy of the fetus showed ectopia cordis and multiple anomalies consistent with amniotic band disruption sequence [Figure 5]. The lower sternum was intact. The diaphragm and pericardium showed no defects. The fetal heart was dissected for further details. The intra-cardiac anatomy diagnosed by fetal echocardiography was confirmed on autopsy [Figure 6]. The atrioventricular orifice was guarded by a common AV valve which was dysplastic.

## DISCUSSION

Prenatal diagnosis of ectopia cordis has been reported rarely in the literature. The condition has a prevalence of 5.5–7.9 per 1 million live births.[1] The most common form of intra-cardiac defects in ectopia cordis include common atrium, AV septal defect, ventricular septal defect, outflow tract anomalies and single ventricle. The fetus in this report had ectopia cordis with common atrium and common AV valve with single ventricle.

Ectopia cordis results from the failure of migration of lateral mesoderm into the midline. The most common associations include sternal defect, pericardial defect, cardiac anomalies and abdominal wall defect, commonly referred to as Pentology of Cantrell. In this fetus, there were no other features of Pentology of Cantrell. Ectopia cordis may be associated with major chromosomal anomalies, especially trisomy 18.[2] The extra-cardiac features in this fetus were consistent with amniotic band disruption sequence. The commonly accepted explanation for amniotic band disruption sequence is the rupture of amnion in early gestation, leading to formation of fibrous bands. These bands cause intrauterine amputations and exteriorization of the viscera. The altered development of thorax prevents the descent of the heart, leading to ectopia cordis, and other mechanical constraints cause cardiac anomalies.[3] To our knowledge, there are no published reports of antenatal diagnosis of ectopia cordis with AV septal defect and single ventricle in association with amniotic band disruption sequence.

The natural course of this condition together with amniotic band disruption sequence is invariably lethal.
